# Outcome of intensive outpatient rehabilitation and bracing in an adult patient with Scheuermann’s disease evaluated by radiologic imaging—a case report

**DOI:** 10.1186/s13013-016-0094-7

**Published:** 2016-10-14

**Authors:** Hagit Berdishevsky

**Affiliations:** Conservative Care for Spine and Scoliosis, ColumbiaDoctors Midtown, Columbia University Medical Center, New York, NY USA

## Abstract

**Background:**

No studies examine the efficacy of intensive specific physical therapy (PT) exercises along with brace for the adult with Scheuermann’s kyphosis (SK).

The aim of this study was to examine the effects of intensive PT based on the Barcelona Scoliosis Physical Therapy School (BSPTS) and SpinoMed brace on a 76-year-old female with SK.

**Case Description:**

A 76-year-old female, diagnosed with SK as an adolescent, presented in October 2014 with thoracic hyperkyphosis T1 to T12 Cobb angle of 85° and lumbar hyper lordosis L1 to L5 Cobb angle of 70°. Lumbar scoliosis T12-L5 with 21° Cobb and vertebral rotation 2. Trunk translation in the sagittal plan was 4.5 cm. Intermittent low back pain 6/10 at worst. Quality-of-life score was 3.8 (SRS 22 questionnaire).

**Method:**

The PT regimen included one-hour Schroth exercise sessions three times per week for 6 months. In addition, a home exercise program (HEP) was recommended. Patient also wore a SpinoMed brace for 2 h per day. All tests and measurements were recorded before and after treatment.

**Results:**

After a six-month treatment period the kyphosis Cobb angle was reduced to 70° and the lordosis Cobb angle improved to 57°. A recent x-ray (October 2015) showed another improvement in the sagittal plane with thoracic kyphosis measuring 64° and lumbar lordosis 55°. Lumbar curvature decreased to 12° and vertebral rotation to 1. The quality-of-life score showed improvement with a score of 4.5 on the SRS 22. Pain score diminished to 2. Trunk deviation improved by 2.2 cm.

**Conclusion:**

These findings suggest that intensive and specific PT and bracing were successful for the treatment of this adult patient with SK.

## Background

Scheuermann’s disease (SD), or Scheuermann’s kyphosis (SK), is a condition developed in the early adolescence in which the normal round-back in the upper spine (called a kyphosis) is increased [1]. This condition has been reported to occur in 0.4 to 8.3 % [OR IS IT 0.8 %??] of the general adolescent population, with an equal distribution between sexes. However, the prevalence and incidence of hyperkyphosis in older adults increases and varies from approximately 20 to 40 % [2]. Most people with SK have an increased round-back (e.g. a hunch back or hump back) but no pain [3]. Nonetheless, untreated kyphosis in the growing child may lead to progressive deformity of the spine and back pain [4].

Several studies report that adolescents with SK who have undergone PT showed improvement in Cobb angle and respiration [5–10]. However, no studies have examined the efficacy of intensive outpatient specific physical therapy exercises for kyphosis along with brace for the management of adults with SK.

The aim of this study was to observe the effect of a six-month intensive physical therapy program based on the BSPTS method – using the Schroth principles and the SpinoMed brace – on the Cobb angle, pain, quality of life, core strength and back strength of a 76-year-old woman with SK.

## Case Presentation

**Fig. 1:**
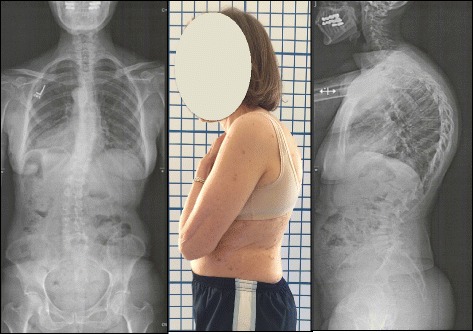
X-ray and clinical picture at evaluation of patient (March 2014): Thoracic kyphosis measured at 86° and lumbar lordosis at 70°

At the time of evaluation, the patient was a 76-year-old woman who was diagnosed with SK when she was an adolescent. Her thoracic kyphosis Cobb angle was measured initially (T1 to T12) at 85° with at least three wedged vertebrae from T5 to T11 (see Fig. 1). Her lumbar lordosis (L1 to L5) was measured with a Cobb angle of 70° (see Fig. 1). Trunk translation in the sagittal plane was 4.5 cm measured T1 to base of S1. The patient also had lumbar scoliosis with convexity on the left side measured at Cobb 21° with vertebral rotation grade 2 according to the Nash and Moe scale [11]. She experienced intermittent low back pain measured at 6/10 at worst using the Visual Analog Scale (VAS). Quality-of-life score was 3.8 using the SRS 22 questionnaire. Core stability and erectors spine strength was graded as good minus (−4/5) using Manual Muscle Testing appropriate tests.

## Method

The PT regimen included one-hour BSPTS/Schroth method exercise sessions that consisted of 1:1 supervised treatments at the patient’s home, three times per week for 6 months, focusing on using the five BSPTS Principles of Corrections (POC) [12] for sagittal plane deformities:

1. **Trunk Elongation** – and expansion throughout the trunk to de-collapse the spine.
2. **Symmetrical Sagittal Straightening** – More specific tension and expansion; in contrast to treatment for scoliosis and other sagittal plane deformities, the correction during this phase is symmetrical for SK, meaning that exercises are identical for both sides of the trunk (right and left):
  - Thoracic expansion bilaterally in the frontal plane and in a posterior to anterior (PA) direction in the sagittal to reduce the hyperkyphosis thoracic;
  - Lumbar expansion bilaterally as well in the frontal plane and in an anterior to posterior (AP) direction in the sagittal plan to reduce the hyperlordotic low back.
3. **Shoulder Traction** (see Fig. 2) – Isometric tension that is performed bilaterally starting at the shoulder region enhances the frontal plane correction/expansion of the thorax.

**Fig. 2:**
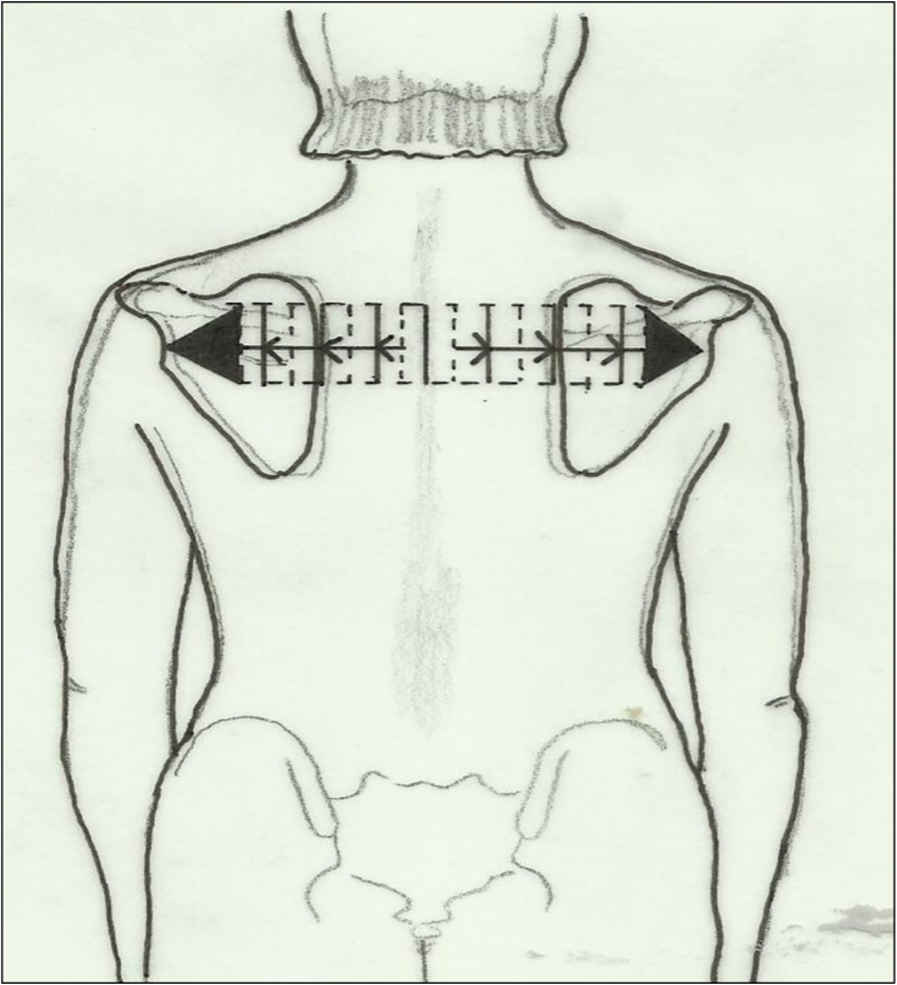
Shoulder Traction - A subject with a hyper-kyphotic morphology will pay more attention to the transversal expansion. Transversal expansion in the proximal thoracic region is an essential part of the correction to facilitate self-elongation (or axial expansion) and can facilitate reduction in the sagittal plane (a plane of maximum deformity in Scheuermann’s kyphosis). *Copyright to Dr. Rigo Manuel*
4. **Corrective Breathing** - Inhaling while maintaining all of the correction principles allows the subject to feel an increased expansion in his/her initially collapsed regions. This is done simultaneously on the right and left sides of the patient with the goal of expanding in a back-to-front direction in the thorax as well as laterally.
5. **Muscle Activation by Increasing Tension** - Isometric tension to achieve the best possible correction and muscle balance. From one side it stabilizes the correction and from the other side it increases the proprioceptive corrective input so that it helps to integrate the ‘corrected body schema’ in the brain.

**Fig. 3:**
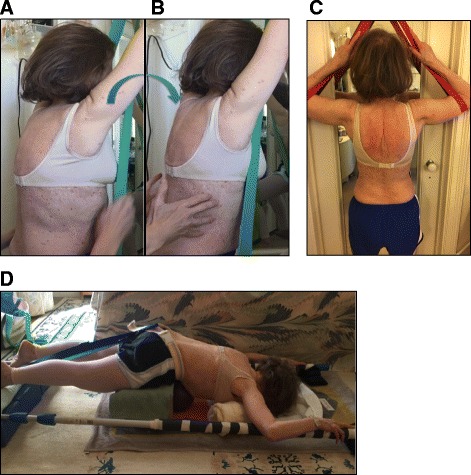
Sample exercises used by patient. From left to right: **a** Modified Semi-hanging starting position and **b** during the activation; **c** Modified “Iguana”; **d** Prone on stool with belt traction

**Fig. 4:**
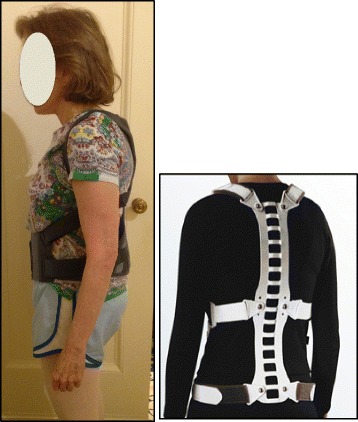
Patient wearing the SpinoMed brace

In addition, a home exercise program, which consisted of 60-minute sessions on a daily basis, was recommended (see Fig. 3 for example of the patient’s home exercises). The patient also wore a SpinoMed brace that was recommended by the therapist, and was instructed to wear the brace while walking for a maximum 2 h each day. The SpinoMed brace is a spinal orthosis that was orignally designed for patients with a diagnosis of osteoporosis [13]. This brace, like other braces for kyphosis, aims to reduce the axial load and shift the center of gravity [14]. It consists of a rigid moldable support contained in a garment that is worn like a backpack and provides support for the lumbar and the thoracic regions of the spine (see Fig. 4). Multiple positive benefits are reported in the literature regarding bracing alone or bracing and exercises for SK [15–18], including a 73 % increase in back extensor strength, 58 % increase in abdominal flexor strength, 11 % decrease in angle of kyphosis, 25 % decrease in body sway, 7 % increase in vital capacity, 38 % decrease in average pain, 15 % increase in well-being, and 27 % decrease in limitations of daily living [13].

During the period of treatment the patient was monitored daily. On days when the therapist was not present, the patient reported which exercises she performed. All tests and and measurements were repeated 6 months after the evaluation.

## Results

**Fig. 5:**
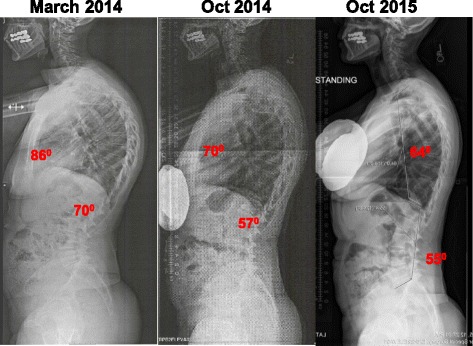
Patient’s before and after x-ras. X-ray on left, taken in March 2014 before beginning therapy showsthoracic kyphosis measuring 86° and lumbar lordosis 70°. X-ray in middle, taken in October 2014, shows patient’s results after 6 months of intensive therapy, with thoracic kyphosis measuring 70° and lumbar lordosis 57°. X-ray on right, taken in October 2015, 1 year after study completed, shows an additional significant decrease in the SK angle with thoracic kyphosis measuring 64° and lumbar lordosis 55°

After a six-month treatment period, the patient was re-evaluated. The patient experienced significant and measurable improvement. The thoracic kyphosis Cobb angle was reduced to 70° and the lumbar lordosis Cobb angle improved to 57°, the lumbar scoliosis curvature decreased to 12° and L3 (lumbar curve apex) vertebral rotation to 1 (according to the Nash and Moe scale of rotation). A second significant improvement was observed on the patient’s recent x-ray (taken in October 2015) with thoracic kyphosis measuring 64° and lumbar lordosis 55° (Fig. 5). The quality-of-life score showed improvement with a score of 4.5 on the SRS 22. Pain score diminished to 0. Trunk deviation improved by 2.2 cm. In addition, a change in the patient’s scoliosis rotation and Cobb angle was observed as a consequence of the exercises. The patient’s core and back extensors strength improved to good plus (+4/5). She reported that she felt more comfortable with her appearance and was satisfied with the results.

## Conclusions

These findings suggest that intensive PT utilizing the BSPTS/Schroth method and bracing was a successful method of treating this adult patient with SK and that patient compliance with home exercises and brace was an important factor contributing to her results.
